# Methane Gas Photonic Sensor Based on Resonant Coupled Cavities †

**DOI:** 10.3390/s19235171

**Published:** 2019-11-26

**Authors:** Carlo Edoardo Campanella, Martino De Carlo, Antonello Cuccovillo, Francesco De Leonardis, Vittorio M. N. Passaro

**Affiliations:** 1 Photonics Research Group, Department of Electrical and Information Engineering, Politecnico di Bari, via E. Orabona n. 4, 70125 Bari, Italy; ce.campanella@qopsys.com (C.E.C.); martino.decarlo@poliba.it (M.D.C.); francesco.deleonardis@poliba.it (F.D.L.); 2 QOpSys s.r.l., 70023 Bari, Italy; an.cuccovillo@gmail.com

**Keywords:** methane gas, photonic sensors, Fabry–Pérot cavity, ring resonators, absorption sensor

## Abstract

In this paper we report methane gas photonic sensors exploiting the principle of absorption-induced redirection of light propagation in coupled resonant cavities. In particular, an example of implemented architecture consists of a Fabry–Pérot (FP) resonator coupled to a fibre ring resonator, operating in the near IR. By changing the concentration of the methane gas in the FP region, the absorption coefficient of the FP changes. In turn, the variation of the methane gas concentration allows the redirection of the light propagation in the fibre ring resonator. Then, the methane gas concentration can be evaluated by analysing the ratio between the powers of two resonant modes, counter-propagating in the fibre ring resonator. In this way, a self-referenced read-out scheme, immune to the power fluctuations of the source, has been conceived. Moreover, a sensitivity of 0.37 ± 0.04 [dB/%], defined as the ratio between resonant modes at different outputs, in a range of methane concentration included between the 0% and 5%, has been achieved. These results allow a detection limit below the lower explosive limit (LEL) to be reached with a cost-effective sensor system.

## 1. Introduction

**Figure 1:**
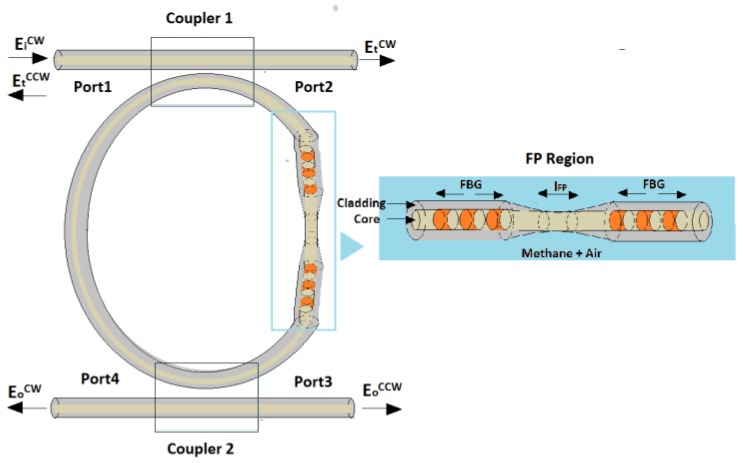
Methane optical sensor based on absorption induced redirection of light propagation in a Fabry–Pérot ring resonator, having clockwise (CW) and counter-clockwise (CCW) exiting optical beams. Inset: sensitive region with length *l_FP_*.

In recent years, optical sensing techniques with coupled resonant cavities have demonstrated several advantages over conventional spectroscopic and interferometric configurations, respectively based on the detection of spectral shift and interference fringe change because of the presence of a measurand. Indeed, physical effects occurring in coupled cavity systems, such as mode splitting, Fano resonance and parity-time (PT) symmetry breaking, have opened new horizons to the optical sensing. If mode splitting-based detection schemes allowed to conceive self-referenced sensing systems (i.e., immune to different sources of noise) [1], the Fano resonance has been exploited to high-resolution methods of transduction [2] while the PT symmetry breaking has been adopted as an efficient way to improve the sensitivity of optical sensors [3]. With the aim of transferring these benefits to a new kind of optical sensors to be used for real life applications, in this paper we propose methane gas sensors based on the redirection of light propagation in coupled resonant cavities, allowing the detection of a methane gas concentration under its lower explosive limit (LEL). This detection feature is crucial in different environments, such as in oil and gas industries and in domestic and commercial sites, where high methane gas concentrations may give rise to an explosion hazard [4]. Being the LEL of methane 5% by volume [5], precise and reliable sensors are needed. To date, catalytic sensors represent the established method for methane gas detection [6,7,8], but they are not methane-specific and do not operate correctly in low-oxygen environments, requiring frequent functional checks. In general, optical sensors can overcome these issues, allowing a fast, selective, non-destructive and intrinsically safe detection. These are the major reasons of the recent interests on portable optical gas sensors [9,10]. Optical gas detection techniques include non-dispersive infrared, spectrophotometry, tunable diode laser spectroscopy and photo-acoustic spectroscopy [11,12]. Among them, we focus on tunable diode laser techniques, exploiting the absorption induced loss, because of the interaction between the evanescent waves and the gas to be measured. Being the direct absorption a weak phenomenon, in order to improve the sensitivity of this kind of sensors it is crucial to enhance the ratio between the power of the evanescent field in the sensing area (where the interaction with the gas occurs) and the total one flowing onto the waveguide, without affecting the intrinsic loss of the sensor. Improvements have been obtained using porous materials or functionalized materials or cladding removal, sol–gel cladding, D-shaped fibres and tapered structures [13]. For hydrocarbon gases, such as methane, the strongest optical absorption occurs in the mid-IR region, around 3.3 µm. However, operating in the mid-IR region has higher costs than the near-IR region and performance limitations [10]. In [14] an evanescent wave sensor with the cladding removal has been proposed for improving the absorption of the methane around the source wavelength of 3390 nm. In [15], a methane sensor, fabricated including cryptophane molecules in the cladding of a polymer-clad silica fibre, has been shown, while in [16] a methane gas sensor with a replacement of the cladding material with a porous one, through the sol–gel technique has been introduced. In both cases, methane sensing relies on the change of the cladding refractive index as function of the gas concentration. In [17], the authors have investigated the shapes of different porous cladding tapered structures, by analysing how the taper ratio influences the time response of these sensors. In the experiments shown in [18], the sensing element for detecting methane concentration consists of a D-shaped fibre, excited with a low cost 1660 nm LED. Through this last sensing solution, it is possible to operate at the absorption peak of the methane gas in the spectral range of the near-IR [19], for which inexpensive light sources and optical detectors are commercially available. In this scenario of evanescent wave sensors operating in the near IR, we propose methane gas sensors based on the absorption induced redirection of light propagation in coupled resonant cavities. These sensors are based on the absorption-induced loss, proper of the evanescent wave sensors, and ruled by the Lambert–Beer law. Their architecture consists of a Fabry–Pérot (FP) resonator coupled to a fibre ring resonator, operating in the near-IR, as reported in Figure 1.

The sensitive region is included between the two mirrors of the FP cavity (i.e., two fibre Bragg grating [20]) and it is supposed to be relied through the cladding and the partial core removal.

## 2. Modelling and Operating Principle

The methane gas optical sensor is based on the optical architecture proposed in [20], consisting of a Fabry–Pérot ring resonator excited at Port 1 through a clockwise (CW) laser source. The amplitude of the CW electrical field component is called *E_i_^CW^* in Figure 1.

As shown in [20], the amplitudes of the output fields, exiting from Port 4 and 3, are associated to the two counter-propagating resonant beams (i.e., the clockwise (CW) and the counter-clockwise (CCW) one), respectively given by:

(1) $$E_{O}^{CW}=\frac{1}{2}Sym+\frac{1}{2}Asym$$

(2) $$E_{O}^{CCW}=\frac{1}{2}Sym-\frac{1}{2}Asym$$

with *Sym* and *Asym* expressed as [20]:

(3) $$Sym=-\frac{k^{2}a^{I}e^{-j\beta L/2}\left(t^{2}ae^{j\frac{\beta l_{FP}}{2}}+r\left(t^{2}-\left(r^{2}+1\right)\right)\right)}{\left(r^{2}-1\right)+\tau^{2}a^{I}{}^{2}e^{-j\beta L}\left(t^{2}ae^{j\frac{\beta l_{FP}}{2}}+r\left(t^{2}-\left(r^{2}+1\right)\right)\right)}E_{i}^{CW}$$

(4) $$Asym=-\frac{k^{2}a^{I}e^{-j\beta L/2}\left(t^{2}ae^{j\frac{\beta l_{FP}}{2}}-r\left(t^{2}-\left(r^{2}+1\right)\right)\right)}{\left(r^{2}-1\right)+\tau^{2}a^{I}{}^{2}e^{-j\beta L}\left(t^{2}ae^{j\frac{\beta l_{FP}}{2}}-r\left(t^{2}-\left(r^{2}+1\right)\right)\right)}E_{i}^{CW}$$

where *β* is the propagation constant of the optical mode in the fibre optic waveguide equal to 2π*n/λ*, where *n* is the group index of the propagating optical mode and *λ* the wavelength; *τ* and *k* the forward-transmitted and cross-coupled optical amplitude coefficients, associated with the two evanescent fibre couplers, easily commercially available as fibre directional couplers [21]; *a^I^* is the attenuation coefficients of the fibre loop, equal to *exp*(−*α^I^L*/2), with *L* fibre loop length and *α^I^* the loss per unit length in the loop; *a* is the attenuation of the Fabry–Pérot, equal to exp(−*αl_FP_*/2), with *l_FP_* as the Fabry–Pérot length and *α* the loss per unit length inside the Fabry–Pérot. The coefficient *t* and *r* are the amplitude reflection and transmission coefficients of the fiber Bragg grating (FBG) mirrors, [2]:

(5) $$\begin{array}{l} r=\frac{K\sin h(\Theta \ell )}{\Theta \cos h(\Theta \ell )+j\Delta \beta \sin h(\Theta \ell )}\\ t=\frac{\Theta }{\Theta \cos h(\Theta \ell )+j\Delta \beta \sin h(\Theta \ell )} \end{array}$$

where *ℓ* is the length of the FBG, while Θ, K and Δβ can be expressed as:

(6) $$\Theta ={\left[{\left|K\right|}^{2}-{\left(\Delta \beta \right)}^{2}\right]}^{\frac{1}{2}};\;K=\frac{\pi \Delta n}{\lambda_{B}}\;;\;\Delta \beta =\beta -\frac{2n\pi }{\lambda_{B}};\;\beta =\frac{2\pi \pi }{\lambda };$$

with Δ*n* and *λ_B_* the index modulation depth and the Bragg wavelength of the FBG. In particular 2*n* *ℓ*/*λ_B_ = M*, with *M* the number of periods, being *Λ_B_ = λ_B_/(*2*n)* the period of the FBG. The amplitude of the coefficient *r* depends on the Bragg wavelength, the number of periods and the index modulation depth, while the transmission *t* can be expressed also as *sqrt* (1 − *r*^2^). The total Fabry Perot length (*l_FP_*) should include also the contribution of the two FBGs (i.e., 2*ℓ*), that can be neglected if *l_FP_ >>* *ℓ*. 

By fixing the mirror reflectivity *r* and the coupling coefficients *k*, the device reported in Figure 1 modulates the intensity of the two counter-propagating optical fields (i.e., clockwise (CW) and counter-clockwise (CCW)), exiting from the FP ring resonator via coupler 2) through the variation of the absorption loss inside the FP [20]. In particular, by properly choosing *r* and *k*, there are values of *a* (i.e., *α*) that can induce a unidirectional backward condition for which the light is routed on the CCW exit (*E_O_^CCW^*), although the excitation occurs through a CW field, called *E_i_^CW^* at port 1 in Figure 1. These values of *a* can be derived by imposing that *Sym = −Asym* at the resonance condition [20]. This physical condition *Sym = −Asym* has been already used to experimentally demonstrate that the anti-symmetric resonance line enhances the light-matter interaction for absorption spectroscopy applications [22].

Differently from [22], the optical sensor reported in Figure 1 is able to redirect the light propagation depending on the variation of *a* (see Equation (6)), with the physical principle reported in [20]. 

Thus, by exciting the CW field *E_i_^CW^* at port 1, we have considered the contrast ratio *C_R_* (defined as in Equation (7)), evaluated at a FP resonance wavelength, as the output of the sensor.

(7) $$C_{R}\left(\alpha \right)=10\log\left\{{{\left|\frac{E_{O}^{CCW}\left(\alpha \right)}{E_{O}^{CW}\left(\alpha \right)}\right|}^{2}|}_{E_{i}^{CW}}\right\}$$

By analysing the ratio of the optical amplitudes of the two counter-propagating beams, the readout of the sensor results to be self-referenced because it does not require a reference signal, similarly to the mode splitting sensors [23]. Moreover, because the power fluctuations of the power source concurrently affect the powers of the two counter-propagating beams, the read-out mechanism is immune to the power fluctuations of the source.

In order to increase the absorption loss (*α*) because of the evanescent field interaction with the surrounding environment, the sensitive region of length *l_FP_* can be obtained through a chemical removal (i.e., etching) of the fibre cladding and core (see the inset of Figure 1), applied to a conventional SMF, realizing a microfiber. In particular, the wet chemical etch-erosion procedure shown in [24] could be easily applied to etch the cladding and partially etch the core of a conventional SMF to obtain the desired microfiber. In this way, the total absorption loss per unit length in the cavity can be expressed as:

(8) $$\begin{array}{l} \alpha \;=\;\alpha_{wg}+\Gamma_{met}(\alpha_{met}) \end{array}$$

where *α_wg_* is the loss attenuation for unit length of the waveguide in the absence of methane gas, Γ*_met_* is the fraction of the evanescent field interacting with the methane gas and *α_met_* is the loss per unit length because of the presence of the methane gas.

In particular, according to the Lambert–Beer law, *α_met_* is proportional to the molarity *M* of the gas (*α_met_* = *εM,* with *ε* the molar absorption coefficient of the methane gas). 

## 3. Design and Optimization

In this section we discuss the sensor design and performance.

In our simulations, the molar absorption coefficient *ε* has been evaluated using the results of [19], where the absorption percentage of the methane gas at 1 atm has been measured in a 60 cm-long cell in the near-infrared region, with a peak centred at 1665.11 nm. In particular, the molarity of the gas has been calculated through the ideal gas law *PV = nRT*.

**Figure 2:**
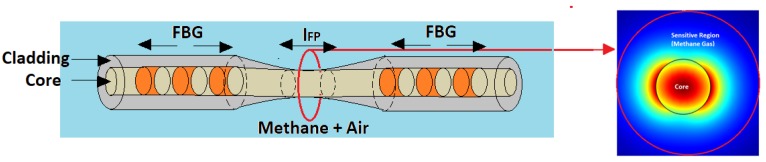
Optical mode in the microfiber computed with COMSOL Multiphysics.

A diameter of the microfiber of 950 nm results to be a good trade-off between the losses of the waveguide in the absence of the methane gas (unperturbed condition) and the ratio of power flowing in the FP region interacting with the methane gas. In particular, we used an *α_core_* = 0.014 dB/cm as the loss of the unperturbed waveguide as in [25] and a Γ*_met_* *=* 51.92% has been evaluated through the finite element method software COMSOL Multiphysics simulations (see Figure 2). In particular, we used the ‘mode analysis’ study on the ‘electromagnetic waves, frequency domain’ physics of the ‘optics’ module of the software.

In order to increase light interaction with methane gas, the length of the FP cavity has been designed to be 80 cm, while the length of the coil has been chosen to be 20 times longer than the FP cavity one. The length of the FP cavity has been designed as a trade-off between the size of the sensor and the need of increasing the absorption path inside the cavity, in order to improve the interaction with the methane gas. 

**Figure 3:**
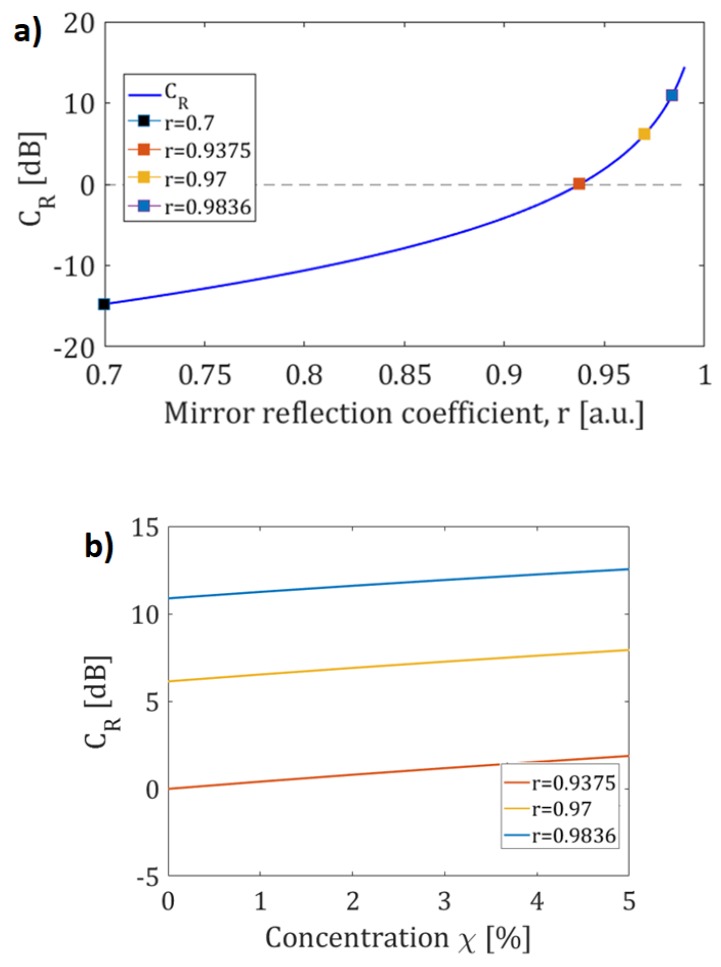
(**a**) *C_R_* as a function of mirror reflection coefficient; (**b**) *C_R_* as a function of the methane gas concentration *χ* for three different values of *r.*

In Figure 3a, the contrast ratio as function of the mirror reflection coefficient has been evaluated in the condition of absence of methane gas, while in Figure 3b the contrast ratio (*C_R_*) as a function of *χ* (being *χ* volume-volume percent concentration). 

**Figure 4:**
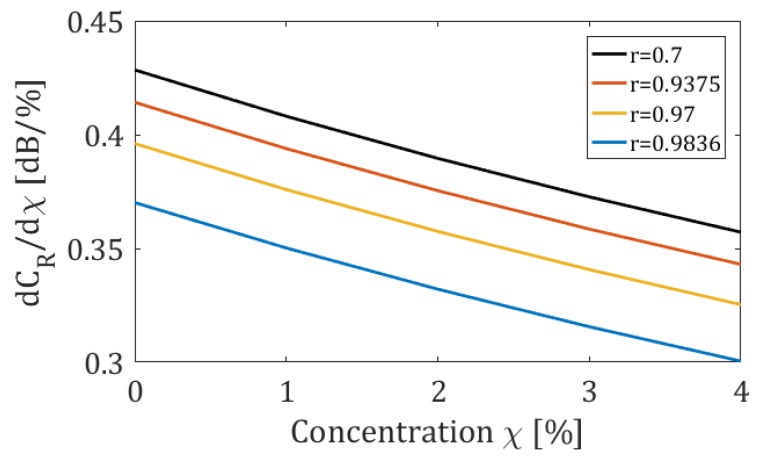
*C_R_* as a function of mirror reflection coefficient (top); sensitivity *dC_R_/dχ* as a function of the methane gas concentration *χ* (bottom).

In Figure 4 (bottom), the sensitivity *d(C_RdB_)/dχ*, as a function of *χ*, is shown for the values of reflection coefficient marked in Figure 3a. It can be observed that for all considered values of reflection coefficient, except for the last one, the sensitivity of the sensor is nearly invariant with *χ*. 

**Figure 5:**
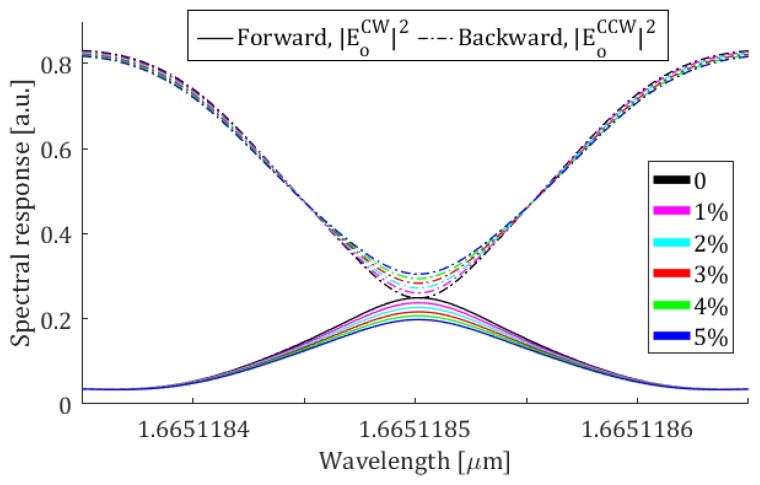
Spectral response for different concentrations of methane gas in the range 1–5% for *r* = 0.9375.

In Figure 5 the spectral response of the sensor for a reflection coefficient of 0.9375 is shown. With reference to Equations (5) and (6), this value of *r* can be obtained with FBG mirrors having a period of 571 nm, Bragg wavelength of 1665.11 nm [19] and length *ℓ* = 9.1 mm, with a moderate modulation depth Δ*n* = 10^−4^ [2], allowing a good tolerance to fabrication errors. The condition of *r* equal to 0.9375 corresponds to an unperturbed *C_R_* equal to 0 dB (same power exiting from the two output ports), leading to a sensitivity of about 0.37 dB/%. As can be observed, by choosing a FBG reflection coefficient higher than 0.9375, the power exiting from the Port 4 is too low, implying a major influence of the electronic readout noise. *C_R_* = 0 dB and *r* = 0.9375 are the optimal design conditions, leading to a good trade-off between the sensitivity and the detection limit, imposed by readout electronics. 

**Figure 6:**
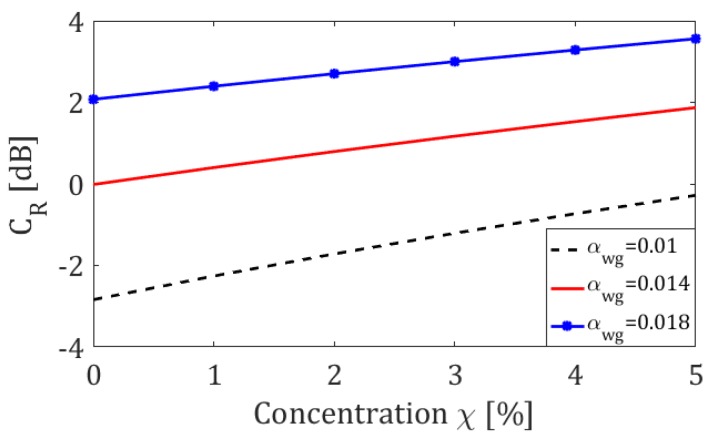
Contrast ratio *C_R_* in dB as a function of methane gas concentration, for different values of attenuation of the microfibre, with FBG reflection coefficient fixed at 0.9375.

The sensor features (*C_R_* versus concentration) can be immediately obtained as shown in Figure 6 (solid red curve). The design curves of the sensor as a function of concentration *χ* have been also evaluated for different values of attenuation of the microfibre *α_wg_*, assuming the FBG reflection coefficient to be 0.9375. In Figure 6 the curves for *α_wg_* = 0.01 (black dashed line) and *α_wg_* = 0.018 (blue starred line) are also reported. As can be easily seen by their comparison, by reducing the attenuation of the microfibre *α_wg_* it is possible to increase the sensitivity of the sensor.

## 4. Conclusions

In this paper we reported a methane gas optical sensor based on the redirection of the light propagation induced by the methane absorption. The sensor has been designed by coupling a Fabry–Pérot cavity with a ring resonator. A change in the absorption coefficient in the FP cavity, induced by a variation in methane concentration, modifies the power exiting from the ports 3 and 4, associated to the two counter-propagating beams. The reflection coefficient of the FBG mirrors of the FP cavity results to be crucial in the design of the sensor. We chose the reflection coefficient of 0.9375, as a good trade-off between the necessity of a high sensitivity, good linear behaviour and high signal to noise ratio at both the output ports.

The contrast ratio *C_R_* (i.e., the ratio between the powers of the light exiting from ports 3 and 4) has been evaluated for different volume–volume percent concentration of methane gas. For values of methane concentration ranging between 0% and 5%, a sensitivity of 0.37 ± 0.04 dB/% has been obtained.

It is worth mentioning that, by considering *C_R_* as the output of the sensor, the readout scheme is self-referenced, so it is immune to the power fluctuations of the source and to other causes of common noise that concurrently affect the two counter-propagating beams. It should be noticed that this sensor is able to detect concentrations of methane gas below its LEL, with a low-cost architecture.

Despite being limited by the intrinsic loss of the microfibre, better performance can be achieved by exploiting the same operating principle with other low loss configurations of coupled optical cavities. This manuscript is an extension version of [26].
